# Supplementary material for: Scale adaptive and robust intrinsic dimension estimation via optimal neighbourhood identification
Source: Sci Rep. 2026 Apr 21;16:18559. doi: 10.1038/s41598-026-48005-4 (PMC13270142; doi:10.1038/s41598-026-48005-4)
Supplement: Supplementary file 1 — Supplementary Information. [file 41598_2026_48005_MOESM1_ESM.pdf]

Supplement to
"Scale adaptive and robust intrinsic dimension estimation via optimal neighbourhood identification"

Antonio Di Noia[1,2], Iuri Macocco[3], Aldo Glielmo[4], Alessandro Laio[5], and Antonietta Mira[2,6]

April 19, 2026

The following sections are devoted to the derivation of theoretical guarantees for the ABIDE estimator introduced in the main paper. Moreover, we briefly show how the methodology can be extended to its Bayesian counterpart. As a proof of concept, we show that GRIDE, another ID estimator recently proposed by Denti et al. (2022), can be improved by selecting optimal neighbourhoods of data points. In principle, this could be done for other ID estimators based on NNs.

S1 Theoretical guarantees

We provide large-sample guarantees regarding the numerical convergence of the proposed algorithm (Proposition S1) and show consistency of the resulting ABIDE estimator (Proposition S2). Moreover, we show asymptotic normality (Proposition S3) of the estimator.

Notations and conventions. Throughout this supplement, we keep the notation introduced in the main text and use the following additional conventions. For events, "a.s." means almost surely. For random sequences, $Z_n = o_P(1)$ means Z_n converges to 0 in probability. The Euclidean norm in $\mathbb{R}^D$ is denoted by $\|\cdot\|_2$, and for a set $A \subset \mathbb{R}^D$ we write $\mathrm{dist}(x, A) := \inf_{y\in A} \|x - y\|_2$. The intrinsic volume measure on M is denoted by Vol_M. For asymptotic statements regarding ABIDE, we write d_n^* in place of d^* to stress dependence on the sample size n. When needed, we explicitly write $k_i^*(d)$ to emphasize dependence of the selected neighbourhood order on the current iterate d.

[1]Seminar for Statistics, Department of Mathematics, ETH Zurich. [2]Faculty of Economics, Euler Institute, Università della Svizzera italiana. [3]Department of Translation and Language Sciences, Universitat Pompeu Fabra. [4]Banca d'Italia. The views and opinions expressed in this paper are those of the authors and do not necessarily reflect the official policy or position of Banca d'Italia. [5]International School for Advanced Studies (SISSA). [6]Department of Science and High Technology, University of Insubria.

Assumption S1. *There exists a d-dimensional C^1 manifold $M \subset \mathbb{R}^D$ with $d < D$ such that*

$$X_i \in M_\epsilon := \{y \in \mathbb{R}^D : \mathrm{dist}(y, M) \leq \epsilon\} \quad a.s.$$

for all $i = 1, \ldots, n$ and some $\epsilon > 0$ small enough.

Assumption S2. *The observations $X_1, \ldots, X_n$ are iid random variables taking values in $\mathbb{R}^D$ and drawn from a common distribution P_X that is absolutely continuous with respect to Vol_M on M.*

We now derive the main results regarding the numerical convergence of Algorithm 1, as well as the consistency and asymptotic normality of the proposed ID estimator. We start with the following proposition regarding the numerical convergence of Algorithm 1.

Proposition S1. *Under Assumptions S1-S2, for every tolerance $\delta > 0$, Algorithm 1 terminates with probability tending to 1 as $n \to \infty$.*

Proof. Let us consider the function $\widetilde{g} : \mathbb{R}_+ \to \mathbb{R}$ defined as

$$d \mapsto \frac{\frac{1}{n}\sum_{i=1}^n k^*_{A,i}(d)}{\frac{1}{n}\sum_{i=1}^n k^*_{B,i}(d)}$$

and note that Algorithm 1 is the fixed point iteration given by

$$d_{m+1} = \frac{\log(\widetilde{g}(d_m))}{\log(g(d_m))} := G(d_m) \tag{S1}$$

where $g : \mathbb{R}_+ \to \mathbb{R}_+$ is the function given by $d \mapsto c_*^{1/d}$ and $G : \mathbb{R}_+ \to \mathbb{R}_+$. The aim is to employ a (version of) the fixed point theorem to show that the sequence of iterations (S1) converges to a fixed point. Let us consider a family of Borel sets $(\mathcal{G}^i_{h_i})_h \subset [0, \infty)$ given by

$$\mathcal{G}^i_{h_i} = \{d \in [0, \infty) : k^*_i(d) = h_i\},$$

note that $(\mathcal{G}^i_{h_i})_{h_i}$ constitutes a partition of $[0, \infty)$. Now, let $d_1 \neq d_2$, clearly in the trivial case where for all $i = 1, \ldots, n$ we have $d_1, d_2 \in \mathcal{G}^i_{h_i}$ then the map G is a contraction mapping because it is constant. On the other hand, when $d_1 \in \mathcal{G}^i_{h_i}$ and $d_2 \in \mathcal{G}^i_{l_i}$ with $h_i \neq l_i$ we have that G is not guaranteed to be a contraction mapping, and we require a different argument. It is not restrictive to write the cumulative intensity on $B(x_i, r_{i,k^*_{A,i}})$ as $\rho_{A,i} V_{i,k^*_{A,i}}$ for some $\rho_{A,i}$, and the cumulative intensity on $B(x_i, r_{i,k^*_{B,i}})$ as $\rho_{B,i} V_{i,k^*_{B,i}}$ for some $\rho_{B,i}$. Note that, by construction, $t_{A,i}(k^*_i) = \tau\, r_{i,k^*_i}$ and $t_{B,i}(k^*_i) = r_{i,k^*_i}$, and, since $k^*_i \leq k_{\max}$, standard NN theory (Lemma 2.2 in Biau and Devroye, 2015) gives $r_{i,k^*_i} \to 0$ in probability. Moreover, by Assumption S1, $\mathrm{Vol}_M(B(x, \tau r))/\mathrm{Vol}_M(B(x, r)) = \tau^d(1 + o(1))$ as $r \to 0$. Therefore, it follows that

$$\frac{\rho_{A,i} V_{i,k^*_{A,i}}}{\rho_{B,i} V_{i,k^*_{B,i}}} = \tau^d \frac{\rho_{A,i}}{\rho_{B,i}}(1 + o_P(1)) = \tau^d \frac{\rho_{A,i}}{\rho_{B,i}} + o_P(1). \tag{S2}$$

Now, since $k^*_{A,i}$ and $k^*_{B,i} - k^*_{A,i}$ are independent by construction, by explicit computation we have that

$$
\begin{aligned}
p_{k^*_{A,i}|k^*_{B,i}}(x|y) &= \frac{p_{k^*_{A,i}}(x) p_{k^*_{B,i}-k^*_{A,i}}(y-x)}{p_{k^*_{B,i}}(y)} \\
&= \frac{y!}{x!(y-x)!} \frac{(\rho_{A,i} V_{i,k^*_{A,i}})^x (\rho_{B,i} V_{i,k^*_{B,i}} - \rho_{A,i} V_{i,k^*_{A,i}})^{y-x}}{(\rho_{B,i} V_{i,k^*_{B,i}})^y} \frac{(\rho_{B,i} V_{i,k^*_{B,i}})^{-x}}{(\rho_{B,i} V_{i,k^*_{B,i}})^{-x}} \\
&= \binom{y}{x} \Big(\frac{\rho_{A,i} V_{i,k^*_{A,i}}}{\rho_{B,i} V_{i,k^*_{B,i}}} \Big)^x \Big(1 - \frac{\rho_{A,i} V_{i,k^*_{A,i}}}{\rho_{B,i} V_{i,k^*_{B,i}}} \Big)^{y-x}.
\end{aligned}
$$

Therefore, using (S2), it follows that

$$
k^*_{A,i} | k^*_{B,i}(d_1) \sim \text{Binomial}\Big(k^*_{B,i}(d_1), \tau^d \frac{\rho_{A,i}}{\rho_{B,i}} \Big),
$$

with probability tending to 1, for some $d_1 \in [0, \infty)$. As a consequence, defining $\widetilde{k}^*(d_1) := \max_{i=1,\dots,n} \{k^*_{B,i}(d_1)\} + 1$, the version of Hoeffding's inequality introduced in Janson (2004) implies that on an event with probability at least $1 - \gamma$,

$$
\Big| \frac{1}{n} \sum_{i=1}^n k^*_{A,i} | k^*_{B,i}(d_1) - \frac{1}{n} \sum_{i=1}^n k^*_{B,i}(d_1) \tau^d \frac{\rho_{i,A}}{\rho_{B,i}} \Big| \le C_\gamma \frac{\sqrt{\widetilde{k}^*(d_1) \sum_{i=1}^n k^*_{B,i}(d_1)^2}}{n},
$$

for every $\gamma > 0$. Combining the inequality in the previous display with the fact that $k^*_i \le k_{max}$ for all $i = 1, \dots, n$, it follows that, with high probability, the average $\frac{1}{n} \sum_{i=1}^n k^*_{A,i} | k^*_{B,i}(d_1)$ lies in a shrinking neighbourhood around its expectation. Now, note that, as $n \to \infty$, by Assumption S2 and the Lebesgue differentiation theorem, $\rho_{A,i} / \rho_{B,i} \to 1$ in probability for all $i = 1, \dots, n$. Thus, recalling that $\tau := g(d_1)$, it follows

$$
\widetilde{g}(d_1) = \frac{\frac{1}{n} \sum_{i=1}^n k^*_{A,i} | k^*_{B,i}(d_1)}{\frac{1}{n} \sum_{i=1}^n k^*_{B,i}(d_1)} = \frac{\frac{1}{n} \sum_{i=1}^n k^*_{B,i}(d_1) \tau^d + o_P(1)}{\frac{1}{n} \sum_{i=1}^n k^*_{B,i}(d_1)} = g(d_1)^d + o_P(1).
$$

Therefore, $\log \widetilde{g}$ possesses the same asymptotic behaviour of $d \log g$, and G converges uniformly in probability to a constant contraction mapping with fixed point equal to d. The desired result follows by employing (a version of) the fixed point theorem (see Kirk and Sims, 2002), which implies that the sequence of iterations (S1) converges to a fixed point with probability tending to 1. □

Since G tends to a constant function, we expect that 2 iterations are enough to obtain stabilization of Algorithm 1 at a given tolerance, and this is confirmed by the numerical experiments presented in the paper. Moreover, we remark that the regularity of the intensity of the Poisson process can improve the numerical convergence of the algorithm.

In other words, when h is locally well approximated by a constant function, termination of Algorithm 1 holds for much smaller sample sizes. Now we derive consistency for the estimator d_n^*.

Proposition S2. *Under Assumptions S1-S2, d_n^* converges in probability to d as $n \to \infty$.*

Proof. Using the arguments given in the proof of Proposition S1, it follows that for any $\epsilon > 0$ and $\delta > 0$, as $n \to \infty$,

$$P\Big(|d_n^* - d| > \epsilon\Big) \leq P\Big(\{|G(d_n^*) + \delta - d| > \epsilon\}\Big) = P\Big(\Big|\frac{\log(\widetilde{g}(d_n^*))}{\log(g(d_n^*))} + \delta - d\Big| > \epsilon\Big)$$
$$= P\Big(\Big|\frac{d\log(g(d_n^*))}{\log(g(d_n^*))} + \delta - d\Big| > \epsilon\Big) + o(1).$$

It suffices to take $\delta \to 0$ and the thesis follows. □

The next Proposition is a qualitative result concerning the asymptotic normality of the proposed estimator.

Proposition S3. *Under Assumptions S1-S2, there exist real constants c, C such that $Z_n = \sqrt{ncI(d_n^*)}(d_n^* - d + C)$ converges in distribution to $Z \sim \mathcal{N}(0,1)$ as $n \to \infty$.*

Proof. Owing to Proposition S1 d_n^* is bounded in probability, therefore, asymptotic normality follows directly from the classical properties of M-estimators (van der Vaart, 2000) combined with the version of the Central Limit Theorem for dependent random variables of Baldi and Rinott (1989). □

Numerical experiments show that the correction constants c, C (due to dependencies and curvature) can be taken equal to 1 with no significant loss of accuracy when approximating the limiting distribution; see Figure S3.

S2 Bayesian version of ABIDE

The Bayesian version of the proposed approach is a straightforward extension of the Bayesian estimator presented in the supplementary material of Macocco et al. (2023). Here we briefly outline it and show how the adaptive version can be obtained. By a conjugacy argument, consider the following prior, $p = \tau^d \sim \text{Beta}(\alpha_0, \beta_0)$, and derive the corresponding posterior distribution

$$p|k_{B,1}, \dots, k_{B,n} \sim \text{Beta}(\alpha, \beta)$$

where $\alpha = \alpha_0 + \sum_{i=1}^n k_{A,i}$ and $\beta = \beta_0 + \sum_{i=1}^n (k_{B,i} - k_{A,i})$. In our adaptive framework, we have instead the following posterior distribution

$$p|k_{B,1}^*, \dots, k_{B,n}^* \sim \text{Beta}(\alpha^*, \beta^*)$$

where $\alpha^* = \alpha_0 + \sum_{i=1}^n k_{A,i}^*$ and $\beta^* = \beta_0 + \sum_{i=1}^n (k_{B,i}^* - k_{A,i}^*)$. Therefore, omitting the conditioning on $k_{B,1}^*, \ldots, k_{B,n}^*$ for notational convenience, the posterior density of the ID, derived by a change of variable argument, is proportional to

$$f_d(x) \propto \tau^{x(\alpha^*-1)}(1-\tau^x)^{\beta^*-1}|\tau^x \log \tau|,$$

where the posterior expectation and the posterior variance can be derived using the well-known fact that for $X \sim \text{Beta}(\alpha, \beta)$ we have

$$\text{E}[X] = \psi_0(\alpha) - \psi_0(\alpha+\beta), \quad \text{Var}[X] = \psi_1(\alpha) - \psi_1(\alpha+\beta),$$

where $\psi_0(z) = \frac{d}{dz} \log \Gamma(z)$ and $\psi_1(z) = \frac{d^2}{dz^2} \log \Gamma(z)$ are the digamma and the trigamma functions respectively. Thus, we obtain the posterior expectation and the posterior variance

$$\text{E}[d] = \frac{\psi_0(\alpha^*) - \psi_0(\alpha^*+\beta^*)}{\log \tau}, \quad \text{Var}[d] = \frac{\psi_1(\alpha^*) - \psi_1(\alpha^*+\beta^*)}{(\log \tau)^2}. \tag{S3}$$

Therefore, adopting a squared error loss function, $\text{E}[d]$ is a proper Bayesian estimator of the ID and, it gives rise to a Bayesian version of Algorithm 1 obtained setting $d_{\text{next}} = \text{E}[d]$. This is named the *Bayesian Adaptive Binomial ID Estimator* (BABIDE). Now we show that BABIDE inherits the theoretical guarantees of ABIDE.

Proposition S4. *BABIDE is asymptotically equivalent to ABIDE.*

Proof. Using the well-known asymptotic approximation of ψ_0 given by

$$\psi_0(z) \sim \log(z) - \sum_{j=1}^{\infty} \frac{\zeta(1-j)}{z^j}$$

where ζ is the Riemann Zeta function, we obtain the asymptotic equivalence

$$\text{E}[d] \sim \frac{\log(\sum_{i=1}^n k_{A,i}^*) - \log(\sum_{i=1}^n k_{B,i}^*)}{\log \tau},$$

which concludes the proof. □

As a consequence of Proposition S4, algorithm termination is naturally inherited by Proposition S1. Moreover, note that statistical guarantees regarding consistency and asymptotic normality can be directly extended to BABIDE by a posterior concentration argument, and using the classical Bernstein–von Mises theorem (Le Cam, 1986) for the posterior distribution.

S3 Adaptive Generalized Ratios ID Estimator

Another approach that fits into the same theoretical framework outlined in the paper is proposed in Denti et al. (2022). Let $r_{i,l}$ be the distance between unit i and its l-th NN according to a selected metric. Then, let n_1, n_2 be two positive integers with $n_2 > n_1$ and define the ratio $\mu_{i,n_1,n_2} = r_{i,n_2}/r_{i,n_1}$ which is a.s. well-defined for continuous data. For discrete data, duplicated points leading to zero values at the denominator of μ_i, need to be removed. The authors derive the density function of μ_{i,n_1,n_2}:

$$f_{\mu_{i,n_1,n_2}}(\mu) = \frac{d(\mu^d - 1)^{n_2-n_1-1}}{\mu^{d(n_2-1)+1}\mathrm{B}(n_2 - n_1, n_1)}, \quad \mu > 1 \tag{S4}$$

where $\mathrm{B}(\cdot,\cdot)$ is the Beta function and d is the ID. In the following, we omit n_1 and n_2 from the subscript for notational convenience. The Authors introduce the *Generalized Ratios ID Estimator* (GRIDE) given by

$$\widehat{d} = \arg\max_{d\geq 0} \log L(d) = \arg\max_{d\geq 0} \log \prod_{i=1}^{n} \frac{d(\mu_i^d - 1)^{n_2-n_1-1}}{\mu_i^{d(n_2-1)+1}\mathrm{B}(n_2 - n_1, n_1)}, \tag{S5}$$

where the maximization is carried out numerically. This approach generalizes the 2NN estimator proposed in Facco et al. (2017), since when $n_1 = 1$ and $n_2 = 2$, the density in (S4) reduces to a Pareto$(1, d)$ and $\widehat{d} = n/\sum_{i=1}^{n} \log \mu_i$, which is equivalent to the estimator proposed in Facco et al. (2017). It must be pointed out that, when compared to the GRIDE estimator based on values of $n_2 > 2$, the 2NN is more likely to satisfy the local homogeneity assumption of the Poisson process density because it only uses information up to the scale of the second NN of each statistical unit. On the other hand, it is potentially affected by the presence of noise in the data. The selection of n_1 and n_2 is somehow linked to the selection of the radii in the framework of Macocco et al. (2023), where the choice of the radii induce $k_{A,1} \ldots, k_{A,n}, k_{B,1} \ldots, k_{B,n}$ in estimator (1), and this choice is affected by the same trade-off between robustness to noise and local homogeneity. The estimator in (S5) is straightforwardly extended to its adaptive version by splitting the likelihood in its single unit contributions, plugging in the unit dependent $n_{2,i} = k_i^*$ and, as suggested in Denti et al. (2022), taking $n_{1,i} = n_{2,i}/2$. This leads to an iterative algorithm similar to ABIDE. We might refer to it as *Adaptive GRIDE* (AGRIDE). Some simulations showed a comparable behaviour of ABIDE and AGRIDE. However, it should be recalled that ABIDE is based on BIDE, which has a closed-form expression and does not require additional numerical routines to be computed. This is a relevant advantage as it makes the adaptive version mathematically more convenient when deriving theoretical guarantees.

S4 Additional experiments

S4.1 Behaviour of adaptive neighbourhoods

Figure S1 reports a comparison of the distributions of k_i^* (left) and corresponding radii $t_{B,i}(k_i^*)$ (right) obtained using the ID estimated with the 2NN estimator (blue) against those obtained with ABIDE (orange) in reference to Panel E of Figure 3. The associated IDs are ≈ 5 for 2NN and ≈ 2.35 for ABIDE. One can appreciate how the neighbourhoods become larger together with the lowering of the ID. This shows how a proper estimation of the ID also affects, non-trivially, the estimation of k_i^* and vice-versa. The right panel displays the distribution of the distances associated with the selected neighbours. One can appreciate how also this distribution is broad, with a long tail and two distinct peaks at small distances. The shape of such distributions allows us to understand why the results obtained at fixed uniform radius $t_{B,i} = t_B$ or at fixed uniform neighbourhood extension $k_i = k$ could not provide reliable results for this kind of dataset, where the density is rapidly changing in a non-uniform way across the dataset.

Figure S2 shows the evolution of $\overline{k^*} = \frac{1}{n}\sum_{i=1}^n k_i^*$ (top) and corresponding $\overline{t_A(k^*)} = \frac{1}{n}\sum_{i=1}^n t_{A,i}(k_i^*)$ (bottom) computed on multivariate Gaussian data. In each panel, the parameters that do not vary are fixed at $n = 10,000$, $d = 5$ and $\alpha = 0.01$. It is at once apparent that $\overline{k^*}$ scales sub-linearly with the sample size (top-left), and $\overline{t_A(k^*)}$ decreases as n increases (bottom-left), making the neighbourhoods asymptotically smaller and smaller. In the central panels, we generate data with different ID values and compute $\overline{k^*}$ and $\overline{t_A(k^*)}$ using the true ID. It is clear that $\overline{k^*}$ decreases as the ID increases since density variations happen at smaller neighbourhoods, while $\overline{t_A(k^*)}$ increases because higher dimensions imply higher distance between data points. Finally, on the right panels, we show that a larger α leads to smaller neighbourhoods ensuring higher protection against type 2 errors.

S4.2 Large sample distribution

Figure S3 reports the Monte Carlo distribution of the centred, re-scaled and normalized statistic $\sqrt{nI(d_n^*)}(d_n^* - d)$ for $n = 10,000$ based on 500 Monte Carlo independent replicas of a 5-dimensional uniform distribution with periodic boundary conditions. From the figure it is at once apparent that the distribution of $\sqrt{nI(d_n^*)}(d_n^* - d)$ tends to be Gaussian, thus confirming the results of Proposition S3.

S4.3 Selection of the testing threshold and results on execution times

One approach to selecting the testing threshold D_{thr} is to use a Bonferroni-adjusted $(1-\alpha)$ quantile of the χ_1^2 distribution. Denoting by h the number of sequential tests performed for each data point, we compare the following four choices: $\alpha = 0.01$, $\alpha = \frac{0.01}{h}$, $\alpha = \frac{0.01}{n}$, and $\alpha = \frac{0.01}{nh}$. We consider the same setting of Figure 2, i.e., points sampled from a 2-dimensional Gaussian distribution, embedded in 100 dimensions with added noise. Results

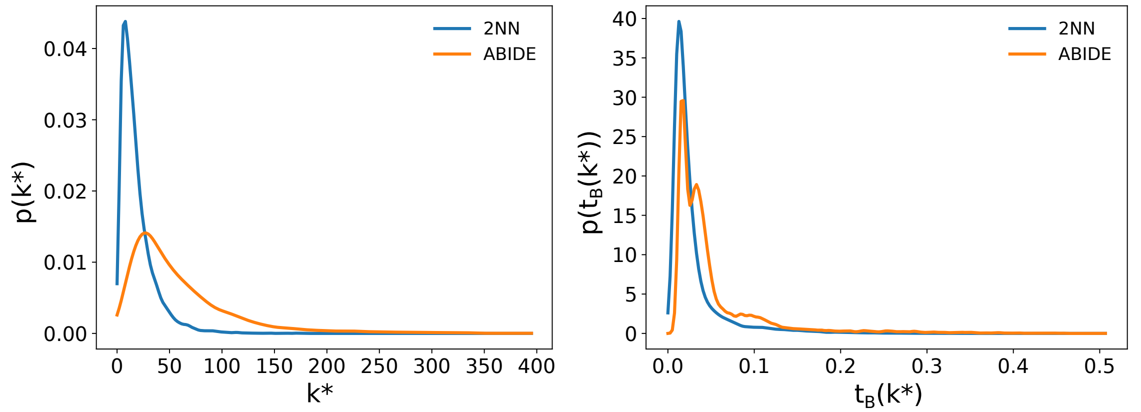

Figure S1: Möbius dataset: comparison of the distributions of k_i^* (left) and corresponding radii $t_{B,i}(k_i^*)$ (right) obtained using the ID estimated with the 2NN estimator (blue) against those obtained with ABIDE (orange) in reference to Panel E of Figure 3.

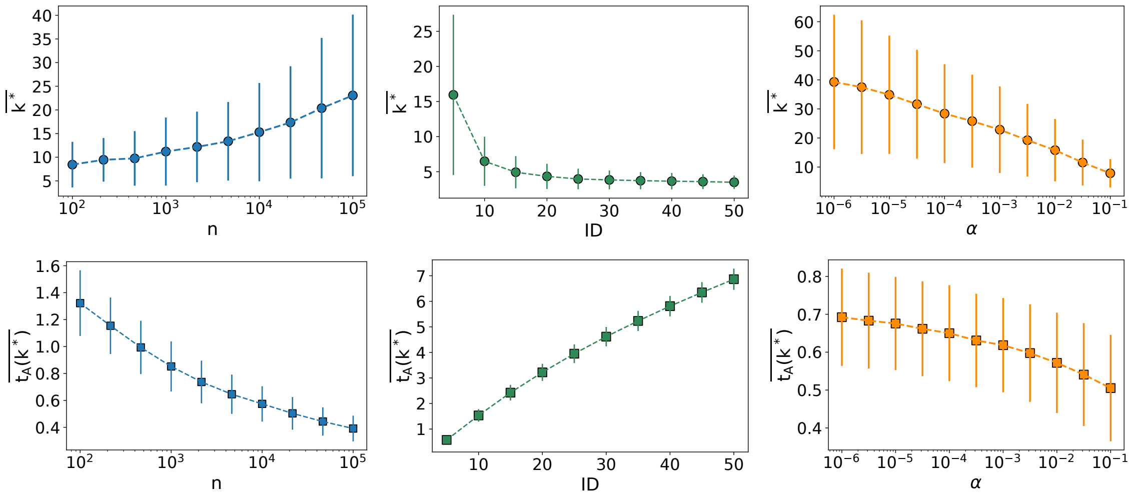

Figure S2: Evolution of $\overline{k^*} = \frac{1}{n}\sum_{i=1}^{n} k_i^*$ (top) and corresponding $\overline{t_A(k^*)} = \frac{1}{n}\sum_{i=1}^{n} t_{A,i}(k_i^*)$ (bottom) computed on multivariate Gaussian data. The vertical bars represent the standard deviation of the distributions. In each panel, the parameters that do not vary are fixed at $n = 10,000$, $d = 5$, and $\alpha = 0.01$.

are reported in Figure S4, where we show the ID estimate as a function of the number of data points n (left), and of the noise intensity (right), for the four different choices of α. In practice, we observe these corrections to be relatively negligible in terms of ID estimates when the sample size is sufficiently large. However, as a general guideline for practitioners, if there is suspicion of substantial noise, we suggest setting a larger threshold D_{thr} which corresponds to a smaller α, e.g. inversely proportional to the number of data points.

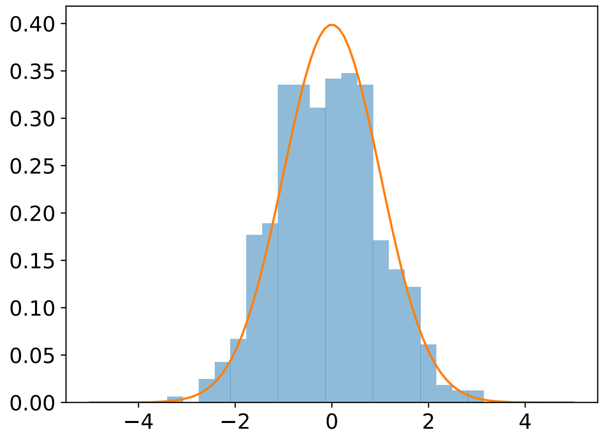

Figure S3: Monte Carlo distribution of the centred, re-scaled and normalized statistic $\sqrt{nI(d_n^*)}(d_n^* - d)$ for $n = 10,000$ based on 500 Monte Carlo independent replicas of a 5-dimensional uniform distribution with periodic boundary conditions.

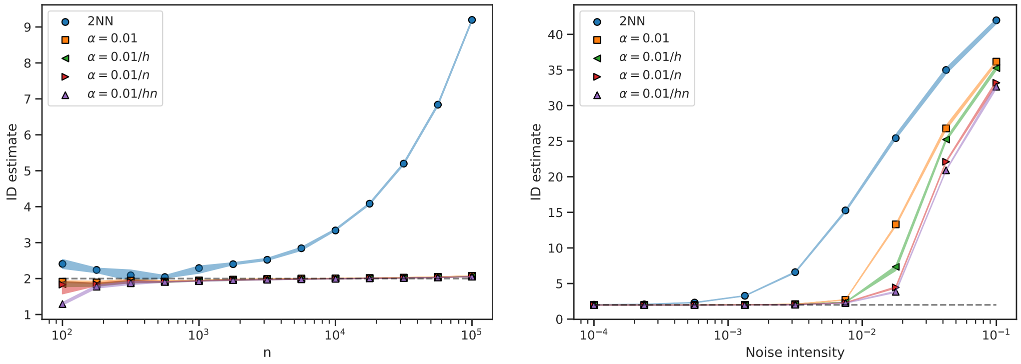

Figure S4: Effect of different Bonferroni correction methods on the ID estimates for a 2 dimensional Gaussian embedded in 100 dimensions. **Left**: ID estimates as a function of the number of data points with the noise fixed to $\sigma_\epsilon = 10^{-3}$. **Right**: ID estimates as a function of the noise with number of point fixed to $n = 5000$.

In Table S1 we report the execution times for the noisy Gaussian distribution (Section 3.1) and for the Möbius strip (Section 3.2). It is evident from Table S1 that the nearest-neighbour computation dominates the execution time, being on average five times slower than the 5-iteration ABIDE workflow.

Dataset ($n \times D$)	NN	$\alpha = 0.01$	$\alpha = \frac{0.01}{h}$	$\alpha = \frac{0.01}{n}$	$\alpha = \frac{0.01}{nh}$
Möbius strip ($20k \times 50$)	5.3	0.22	0.27	0.36	0.51
Gaussian ($5k \times 100$)	2.5	0.10	0.11	0.12	0.17

Table S1: Running times (in seconds) across two datasets for a single i7-1165G7 core. The values reported in the columns with α are intended for a single iteration step, comprehensive of both k^* and ID calculation.

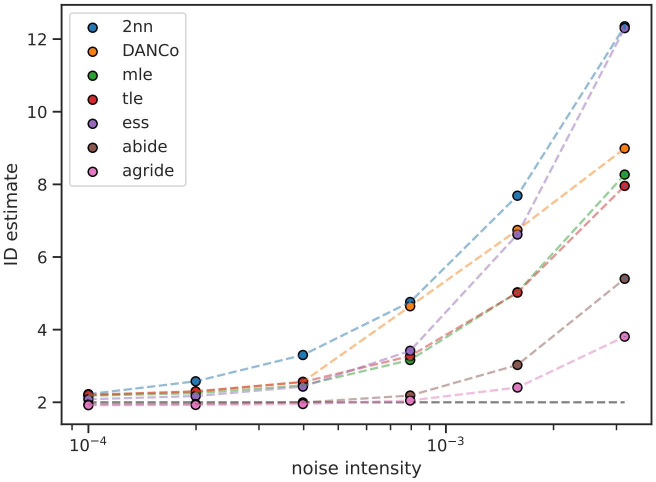

Figure S5: ID estimates as a function of the noise added to a 2-dimensional manifold twisted and embedded in 50 dimensions. We report the results for the most commonly used NN-based ID estimators.

S4.4 Comparison with other NN-based ID estimators

We compare the performance of both ABIDE and AGRIDE against the other most used kNN estimators, namely DANCo (Ceruti et al., 2014), MLE (Levina and Bickel, 2004), TLE (Amsaleg et al., 2019), and ESS (Johnsson et al., 2014). We reproduce the experiment of Panel F of Figure 3, namely a 2-dimensional, highly non-uniform manifold, which is twisted and then embedded in 50 dimensions. The noise is then added to all the 50 dimensions. The experiments were carried out using the Scikit-dimension (Bac et al., 2021) package, setting the number of considered neighbours to 20. From Figure S5, ABIDE and AGRIDE are manifestly superior in resisting to the noise and, thus, providing a better ID estimate.

References

Amsaleg, L., Chelly, O., Houle, M. E., Kawarabayashi, K.-I., Radovanović, M., and Treeratanajaru, W. (2019). Intrinsic dimensionality estimation within tight localities. In

Proceedings of the 2019 SIAM international conference on data mining, pages 181–189. SIAM.

Bac, J., Mirkes, E. M., Gorban, A. N., Tyukin, I., and Zinovyev, A. (2021). Scikit-Dimension: A Python Package for Intrinsic Dimension Estimation. *Entropy*, 23(10):1368.

Baldi, P. and Rinott, Y. (1989). On normal approximations of distributions in terms of dependency graphs. *The Annals of Probability*, 17(4):1646–1650.

Biau, G. and Devroye, L. (2015). *Lectures on the nearest neighbor method*, volume 246. Springer.

Ceruti, C., Bassis, S., Rozza, A., Lombardi, G., Casiraghi, E., and Campadelli, P. (2014). Danco: An intrinsic dimensionality estimator exploiting angle and norm concentration. *Pattern recognition*, 47(8):2569–2581.

Denti, F., Doimo, D., Laio, A., and Mira, A. (2022). The generalized ratios intrinsic dimension estimator. *Scientific Reports*, 12(1):20005.

Facco, E., D'Errico, M., Rodriguez, A., and Laio, A. (2017). Estimating the intrinsic dimension of datasets by a minimal neighborhood information. *Scientific Reports*, 7(1):1–8.

Janson, S. (2004). Large deviations for sums of partly dependent random variables. *Random Structures & Algorithms*, 24(3):234–248.

Johnsson, K., Soneson, C., and Fontes, M. (2014). Low bias local intrinsic dimension estimation from expected simplex skewness. *IEEE transactions on pattern analysis and machine intelligence*, 37(1):196–202.

Kirk, W. A. and Sims, B. (2002). Handbook of metric fixed point theory. *Australian Mathematical Society GAZETTE*, 29(2).

Le Cam, L. (1986). *Asymptotic methods in statistical decision theory.* Springer Science & Business Media.

Levina, E. and Bickel, P. (2004). Maximum likelihood estimation of intrinsic dimension. *Advances in neural information processing systems*, 17.

Macocco, I., Glielmo, A., Grilli, J., and Laio, A. (2023). Intrinsic dimension estimation for discrete metrics. *Phys. Rev. Lett.*, 130:067401.

van der Vaart, A. W. (2000). *Asymptotic Statistics*, volume 3. Cambridge University Press.
